# 

**Published:** 1892-09-03

**Authors:** 


					.price twopence.
W, Vol. XII.?Sept. 3rd, 1892.
Hjjtered at the General Post Office as a Newspaper for ;\ , .
'Mumiasion in the United Kingdom and Abroad.] [1 j ?
No. 310.J Vol. XII. J Saturday,
September 3rd, 1892.
[Twopence.

				

